# Iron‐Catalyzed Carbonylation Reactions with Carbon Monoxide

**DOI:** 10.1002/anie.202512346

**Published:** 2025-08-10

**Authors:** Kangkang Sun, Guo‐Ping Lu, Wei Han, Matthias Beller

**Affiliations:** ^1^ State Key Laboratory of Microbial Technology Jiangsu Collaborative Innovation Center of Biomedical Functional Materials Jiangsu Key Laboratory of New Power Batteries School of Chemistry and Materials Science Nanjing Normal University Nanjing 210023 P.R. China; ^2^ Applied Homogeneous Catalysis Leibniz Institute for Catalysis Albert‐Einstein‐Straße 29a 18059 Rostock Germany; ^3^ School of Chemistry and Chemical Engineering Nanjing University of Science and Technology Xiaolingwei 200 Nanjing 210094 P.R. China

**Keywords:** Carbon monoxide, Carbonyl compounds, Carbonylation, Iron, Synthesis

## Abstract

The utilization of carbon monoxide (CO) as an inexpensive and indispensable C_1_ feedstock for the efficient construction of diverse carbonylated compounds has been a subject of numerous studies for decades. Nowadays, transition metal‐catalyzed carbonylation represents a pivotal synthetic approach for the generation of carbonyl‐containing molecules. Among the known catalysts for these transformations, non ‐noble metal‐based systems have garnered increasing interest in recent years due to their substantial advantages, including wide availability, low cost, and low toxicity. In this review, we have summarized and discussed original and recent developments in iron‐catalyzed carbonylative transformations, wherein CO is utilized as the C_1_ feedstock.

## Graphical abstract

Recent developments in iron‐catalyzed carbonylative transformations using CO as a C₁ feedstock are summarized in this minireview. Reaction mechanisms of selected reactions and the potential for further advancements in this field are also discussed.

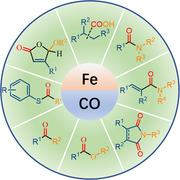

## 1 Introduction

**Figure 1:**
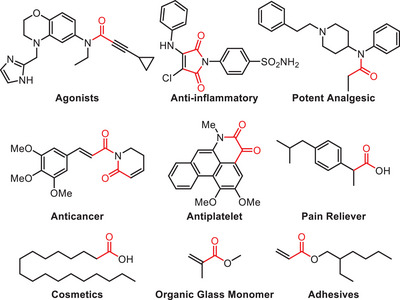
Selected examples of important carbonyl compounds with a focus on biologically active molecules accessible via carbonylation reactions.

Carbonylation is an essential and useful toolbox of reactions for synthesizing a variety of carbonyl‐containing compounds and their derivatives.^[^ ^1^ ^]^ Carbonyl compounds, e.g., esters, amides, ketones, etc., are found everywhere: from daily life chemicals to natural products and large‐scale industrial products (Figure 1). In the chemical laboratories worldwide, they serve as pivotal structural components in numerous organic reactions.^[^ ^2^, ^3^, ^4^, ^5^, ^6^ ^]^ Notwithstanding its substantial industrial applications, synthetic organic chemists are reluctant to employ carbon monoxide (CO) as a one‐carbon feedstock in carbonylation reactions. This is in part due to its gaseous nature and the challenges associated with its handling as well as its toxicity.^[^ ^7^ ^]^ However, its abundance, availability, and low cost make this C_1_ feedstock highly attractive for practical applications.^[^ ^8^, ^9^, ^10^, ^11^, ^12^ ^]^ In the future, we can expect to use biomass gasification,^[^ ^13^, ^14^ ^]^ carbon dioxide reduction with renewable hydrogen,^[^ ^15^, ^16^, ^17^, ^18^ ^]^ and steelmaking tail gas recycling as ways to get CO in a sustainable way.^[^ ^19^ ^]^ At the same time, the global demand for CO is expected to rise, mainly due to increased investment in developing advanced and technical products. More specifically, the CO market is expected to grow from USD 3.50 billion in 2024 to USD 4.93 billion by 2032, with a CAGR of 4.4% during the forecast period.^[^ ^20^ ^]^ Consequently, conversion of CO molecules into value‐added bulk and fine chemicals through carbonylation reactions will continue to be of immense significance.

Recent examples of utilizing transition metal‐catalyzed carbonylation reactions with CO^[^ ^21^, ^22^, ^23^, ^24^, ^25^, ^26^ ^]^ include preparing aldehydes,^[^ ^27^ ^]^ ketones,^[^ ^28^ ^]^ carboxylic acids,^[^ ^29^ ^]^ esters,^[^ ^30^ ^]^ and amides.^[^ ^31^ ^]^ It is worth noting that the majority of catalyst research for carbonylation reactions has centered on the use of precious metal catalysts, such as Ru, Rh, Ir, Pd, and Pt complexes.^[^ ^32^, ^33^, ^34^, ^35^, ^36^ ^]^ Palladium complexes, in particular, have been extensively explored in carbonylation reactions for the formation of carbonyl compounds.^[^ ^37^, ^38^, ^39^, ^40^, ^41^ ^]^ However, the increasingly high price, poor stability, and biological toxicity of noble metals hinder their future applications.^[^ ^42^ ^]^ From the viewpoint of green and sustainable chemistry, the application of non‐noble metal complexes such as Mn,^[^ ^43^ ^]^ Fe,^[^ ^44^ ^]^ Cu,^[^ ^45^ ^]^ Co,^[^ ^46^ ^]^ and Ni^[^ ^47^ ^]^ instead of precious‐metal‐based catalysts is much more desirable. In this respect, especially iron‐catalyzed carbonylation reactions have attracted more and more attention.

Iron is the most cost‐effective and abundant metal, comprising approximately 5% of the Earth's crust. Furthermore, iron displays a wide range of formal oxidation states, spanning from −II to +VI, making it a valuable element in both reductive and oxidative chemical processes.^[^ ^48^, ^49^, ^50^, ^51^ ^]^ Of particular significance is the fact that this metal, in conjunction with its oxides and various salts,

Manifests notable nontoxic properties. Currently, regulatory authorities categorize iron as a “metal with minimal safety concern,” allowing for residual iron levels of up to 1300 ppm in pharmaceutical products.^[^ ^52^ ^]^ Consequently, iron catalysis has the potential to establish a responsible framework for carbonylation reactions and the synthesis of carbonyl‐containing products, while simultaneously offering the prospect of economic benefits. A survey of the existing literature reveals the majority of reviews focus on carbonylation reactions catalyzed by Au, Pd, Ni, Co, or Cu.^[^ ^53^, ^54^, ^55^, ^56^, ^57^, ^58^, ^59^ ^]^ To the best of our knowledge, there is currently no review available that focuses on iron‐catalyzed carbonylation reactions. Thus, through this review, we aim to summarize and discuss relevant literature concerning iron‐catalyzed carbonylation reactions utilizing CO as a C_1_ feedstock. Additionally, we will provide brief discussions on the reaction mechanisms of selected reactions and the potential for further advancement in this field. The following sections are ordered according to the obtained products.

## 2 Iron‐Catalyzed Carbonylation Reactions to Synthesize Esters

Carboxylic esters are of paramount importance in chemistry and biology. Many such compounds are found in natural products and daily‐life chemicals. For example, they are responsible for the fragrance and flavor of flowers and fruits. In addition, main components of our food (glycerides) and many pharmaceuticals, such as clopidogrel, methylphenidate, fenofibrate, prasugrel, oseltamivir, eszopiclone, and fluticasone, contain ester linkages.^[^ ^60^ ^]^ Moreover, they find applications as solvents, softening agents for resins, and key constituents of polymers (polyesters). Therefore, the development of versatile synthetic methods for the preparation of esters remains an important research topic in organic chemistry. Among the many known procedures, the so‐called alkoxycarbonylation or hydroesterification method, which utilizes CO as the carbonyl source, provides a straightforward and atom‐efficient strategy for synthesizing esters. In this section, we will present a summary of protocols for obtaining carboxylic esters through iron‐catalyzed processes utilizing carbon monoxide.

**Scheme 1:**
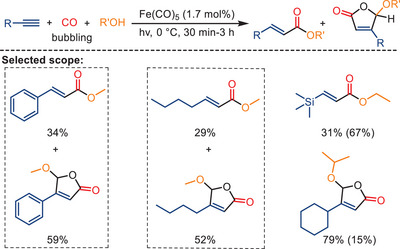
Photochemical iron‐catalyzed formation of *α,β*‐vinylesters and *γ*‐lactones from alkynes under UV irradiation (125 W immersion mercury lamp). Yields of co‐produced lactone or ester are shown in parentheses. 14 examples examined; ester yields: 8%–37%; lactone yields: 52%–88%.

The first iron‐mediated/catalyzed carbonylation reactions were described by Reppe and his collaborators in the BASF laboratories in Germany from 1938 to 1945. At that time several metal carbonyl‐catalyzed reactions were discovered but remained unpublished until the end of the Second World War.^[^ ^61^ ^]^ Based on the better activity and selectivity of noble metal‐based carbonylation reactions, studies in the coming decades were scarce.^[^ ^62^, ^63^, ^64^ ^]^ The transition of academic interest in homogeneous catalysis development toward 3d metals has also led to a resurgence of interest in this field. As an example, in 2010, Mathur and his colleagues developed a one‐pot photochemical synthesis of *α,β*‐vinylesters and alkoxy‐substituted *γ*‐lactones in the presence of Fe(CO)_5_ (Scheme 1).^[^ ^65^ ^]^ In their work, various terminal acetylenes, specifically ferrocenyl‐, phenyl‐, trimethylsilyl‐, hexyl‐, and cyclohexyl‐substituted acetylene, were employed as substrates in conjunction with alcohols (methanol, ethanol, and isopropanol). In the presence of a catalytic amount (1.7 mol%) of Fe(CO)_5_ and by continuous bubbling of CO through the system, high yields of the desired products were obtained in a single step. Simultaneously, the formation of a trace amount of diphenylquinone by‐products was observed. The selectivity of the products is influenced by both the UV irradiation time and the choice of solvent. As the reaction time increases, the yield of lactones rises, while that of esters decreases. Furthermore, the yield of esters exhibits variations among the alcohols, with the order being methanol > ethanol > isopropanol.

**Scheme 2:**
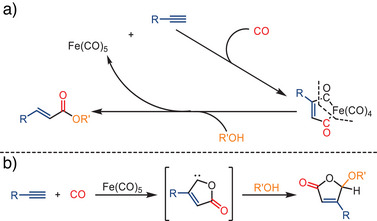
Mechanism for the photochemical iron‐catalyzed formation of *α,β*‐vinylesters and *γ*‐lactones from alkynes.

Mechanistic insights indicate that the reaction of ferrocenylacetylene with Fe(CO)_5_, CO, and alcohols proceeds via a ferrole‐type intermediate, [Fe{C(O)C(H)C(Fc)C(O)}(CO)_4_], which undergoes nucleophilic attack by alcohols to generate vinyl esters (Scheme 2a). Under visible‐light irradiation, the pathway shifts toward the formation of diferrocenyl quinone, while alkoxy‐substituted lactones are proposed to arise via a photoinduced carbene intermediate, as suggested by prior literature (Scheme 2b). Although the ferrole intermediate has only been directly observed in the ferrocenyl system, analogous mechanisms may be relevant for other terminal alkynes under similar conditions.

**Scheme 3:**
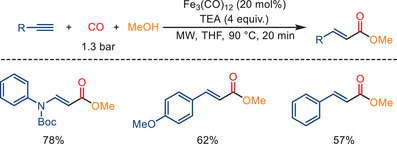
Iron‐catalyzed monocarbonylation of terminal alkynes using microwave irradiation. Three examples examined; yields: 57%–78%.

One year later, Pizzetti and colleagues reported a microwave‐assisted catalytic alkoxycarbonylation of alkynes and methanol, as depicted in Scheme 3.^[^ ^66^ ^]^ Using [Fe_3_(CO)_12_] as the catalyst precursor and triethylamine as the sole ligand, they developed an atom‐economic method for carbonylation of alkynes using low pressures of CO gas (1.3 bar). The implementation of microwave irradiation in these reactions has been shown to simplify the reaction conditions in comparison to the use of classical autoclaves. This has resulted in significant reductions in reaction time (down to 20 min), temperature, and gas pressure. The utilization of methanol as a nucleophilic agent facilitates the transformation of ynamides and alkynes into the corresponding acryl esters, yielding a moderate yield.

**Scheme 4:**
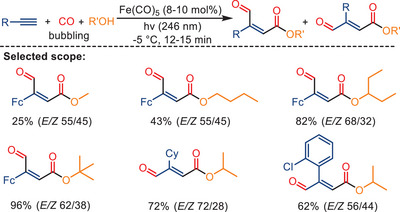
Photochemical reaction of ethynylferrocene with methanol and carbon monoxide in the presence of Fe(CO)_5_ catalyst. 20 examples examined; yields: 25%–96%.

In 2017, Joshi and Satrawala published an intriguing alkoxycarbonylation protocol, wherein they employed iron catalysts to achieve a high‐yield, one‐pot synthesis of biologically significant retinoid esters (Scheme 4).^[^ ^67^ ^]^ Their study revealed that various monometallic carbonyl complexes, including Ru(CO)_5_ and M(CO)_6_ (M = Cr, Mo, W), as well as multimetal carbonyl complexes M_3_(CO)_12_ (M = Ru, Os, and Fe), were incapable of synthesizing the desired product. Iron pentacarbonyl [Fe(CO)_5_] is the most efficacious catalyst for synthesizing *E/Z* alkyl 3‐formyl‐3‐alkyl/aryl/ferrocenyl‐2‐propenoate through the photolysis of terminal acetylenes, alcohols, and CO. The yield of the products and their *E/Z* ratio were significantly influenced by the choice of alcohols utilized as solvents. Specifically, when primary alcohols were used, the yield of retinoid esters consistently increased with the length of the alcohol's carbon chain. When secondary alcohols were employed as solvents, high product yields ranging from 76% to 87% were observed. Even utilizing sterically bulky tert‐butyl alcohol, almost complete conversion of acetylene to the corresponding retinoid esters was achieved. This notable transformation of retinoid esters is attributed to the increased acidity of tertiary alcohols in comparison to secondary and primary alcohols. Overall, twenty distinct retinoid esters were successfully synthesized from readily available alkynes and alcohols by this convenient method. The observation of simultaneous formylation and esterification of terminal acetylene is a noteworthy finding of this study.

**Scheme 5:**
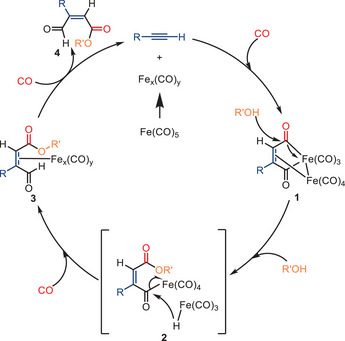
Proposed mechanism for the synthesis of retinoid esters.

After conducting several control reactions, the authors have proposed a plausible catalytic cycle for this transformation (Scheme 5).^[^ ^68^, ^69^ ^]^ Consistent with the findings reported in previous literature, intermediate **1** is initially formed. Subsequently, the nucleophilic attack of the alcohol on the carbonyl carbon results in the formation of intermediate **2**. This intermediate **2** then undergoes a transformation into a significantly more stable intermediate **3** through a hydride transfer from the metal to the carbonyl group. Intermediate **3** then forms the desired *E/Z* alkyl 3‐formyl‐3‐alkyl/aryl/ferrocenyl‐2‐propenoate **4** via a non‐reductive elimination process, while simultaneously regenerating the Fe*_x_* (CO)*_y_* catalyst for subsequent catalytic cycles.

**Scheme 6:**
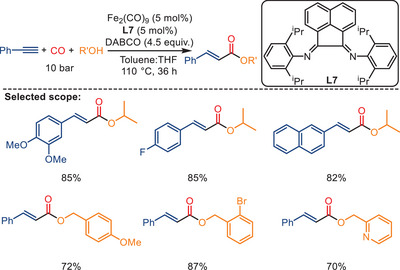
Iron‐catalyzed highly selective hydroalkoxycarbonylation of alkynes. 40 examples examined; yields: 4%–100%.

In 2023, the group of Tewari disclosed an iron‐catalyzed highly selective hydroalkoxycarbonylation of alkynes using CO as a C_1_ feedstock (Scheme 6).^[^ ^70^ ^]^ Using [Fe_2_(CO)_9_] as the catalyst precursor, 1,4‐diazabicyclo[2.2.2]octane (DABCO) as the base, and diimine ligand **L7** as the only ligand, alkynes and alcohols can be transformed into their corresponding *α,β*‐unsaturated esters in one step under 10 bar CO pressure. Mechanistic studies and control experiments suggest that DABCO is essential for this transformation, as it not only facilitates the generation of an alkoxide ion to attack the metal acyl intermediate but also acts through its conjugate acid [DABCO‐H]⁺ to promote the formation of iron hydride species, as supported by NMR evidence. The generality and practicability of the method were impressively demonstrated by synthesizing around 40 *α,β*‐unsaturated esters, including the preparation of important daily life products such as flavors, sunscreen, and antifungal agents on a 1 g scale.

**Scheme 7:**
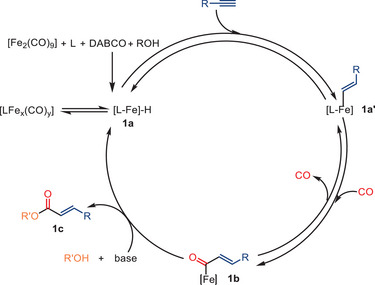
Proposed mechanism for the Fe‐catalyzed hydroalkoxycarbonylation of terminal alkyne.

To understand the catalytic process in more detail, radical trapping and EPR experiments were performed, both suggesting that radical intermediates are not involved in this reaction. Based on a series of additional control experiments and previous research, the authors suggest the plausible mechanism shown in Scheme 7.^[^ ^71^, ^72^, ^73^ ^]^ Initially, the formation of a ligand‐coordinated iron hydride intermediate is proposed. Then, intermediate **1a’** is produced as the alkyne coordinates with the metal and formally inserts into the Fe─H bond in a syn fashion, which accounts for the observed high stereoselectivity in the formation of (*E*)‐configured *α,β*‐unsaturated esters. Next, CO insertion occurs, resulting in the formation of the metal acyl species **1b**. Finally, the desired product, *α,β*‐unsaturated ester **1c**, is generated through the nucleophilic attack of an alcohol. Interestingly, only monocarbonylation products were obtained in this system. Although the authors did not comment on the origin of this selectivity, it is conceivable that the steric bulk of the bisimine ligand **L7** might hinder multiple CO insertions, thus favoring a single‐carbonyl incorporation.

**Scheme 8:**
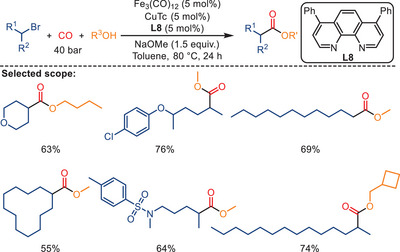
Bimetallic Fe‐/Cu‐catalyzed alkoxycarbonylation of unactivated alkyl bromides. 28 examples examined; yields: 23%–85%.

In addition to the previously documented transformations of alkynes, a variety of carbonylations of C─X bonds (X = Br, I) have been reported in the presence of iron carbonyl complexes. The requisite preliminary materials, halogenated organic compounds, are prevalent intermediates in the synthesis of various carbonyl‐containing molecules.^[^ ^74^, ^75^ ^]^ For instance, in 2018, Wu and Li reported a copper/iron co‐catalyzed alkoxycarbonylation of unactivated alkyl bromides into the corresponding aliphatic esters employing alcohols as the reaction partners (Scheme 8).^[^ ^76^ ^]^ More specifically, in the presence of copper(I) thiophene‐2‐carboxylate (CuTc) and Fe_3_(CO)_12_ using bathophenanthroline (**L8**) as the ligand and NaOMe as the base under 40 bar CO, the corresponding esters were obtained in moderate‐to‐good yields. NaOMe facilitates the generation of the active catalytic species and neutralizes the HBr formed during the reaction. Simple short‐ and long‐chain as well as cycloalkyl bromides yielded the desired products in good yields. Remarkably, the presented bimetallic catalyst can be employed for the alkoxycarbonylation of primary, secondary, and tertiary alkyl bromides. Interestingly, the carbonylation of secondary alkyl bromides is faster than that of tertiary alkyl bromides. Regarding the alcohol, primary alcohols gave high yields of the desired esters. Secondary alcohols were also effective in this protocol; however, the yield of the corresponding ester product decreased. As expected, tertiary alcohols, such as tertiary butanol, gave a very low yield of the desired product.

**Scheme 9:**
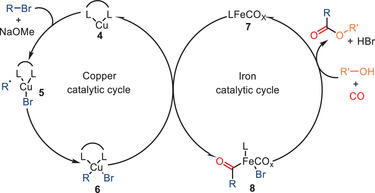
Proposed reaction mechanism for Fe/Cu co‐catalyzed carbonylative transformation of alkyl bromides.

In addition to the optimization of the catalyst system and the investigation of the protocol's scope, the authors proposed a tentative mechanism based on control experiments (Scheme 9). Firstly, NaOMe assists the copper complex **4** in irreversibly abstracting a bromine atom from the alkyl bromide, producing a carbon‐centered radical and a copper bromide intermediate **5**. Subsequently, a radical addition to copper complex **5** occurs, resulting in the formation of the new copper complex **6**. Following this, intermediate **6** reacts with iron complex **7**, forming the acylcarbonyl‐iron complex **8** through transmetalation and CO insertion steps. The desired ester product is ultimately obtained through the nucleophilic attack of the alcohol, which concurrently regenerates the active iron species **7**.

**Scheme 10:**
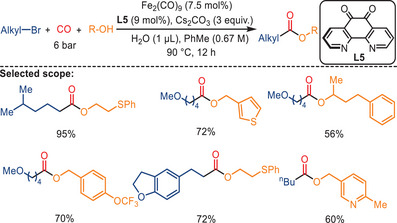
Fe‐catalyzed alkoxycarbonylation of unactivated alkyl bromides with alcohols. Over 80 examples examined; yields: 44%–95%.

Subsequent to these initial findings, the same group published a report four years later detailing the iron‐catalyzed alkoxycarbonylation of alkyl bromides for the synthesis of esters via a two‐electron transfer process (Scheme 10).^[^ ^77^ ^]^ In the presence of the Fe_2_(CO)_9_, **L5**, and Cs_2_CO_3_ under 6 bar of CO pressure, good‐to‐excellent yields of the target products (up to 95%) were achieved. In this advanced protocol water functions as a polar protonic reagent, thereby enhancing the solubility of the Cs_2_CO_3_ and facilitating the nucleophilic attack and reductive elimination steps. This, in turn, increases the reaction's reactivity. It is noteworthy that the valence state of the iron sources plays a pivotal role in the initiation of the reaction. Specifically, only Fe(0) and Fe(−II) sources were found to be capable of initiating the reaction, while no alkoxycarbonylation was observed when Fe(II) or Fe(III) catalysts were employed. This method demonstrated compatibility with a wide array of functional groups and structural blocks. In addition to alkyl bromides, other unactivated alkyl electrophiles, including alkyl iodides, tosylate, and mesylate, could also be utilized to synthesize the desired esters.

**Scheme 11:**
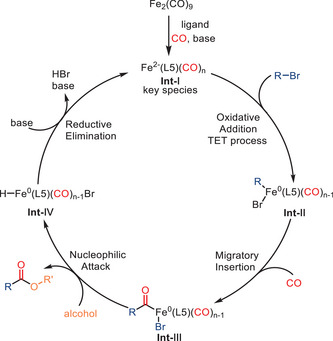
Proposed mechanism for the alkoxycarbonylation of alkyl bromides.

In the proposed mechanism (Scheme 11), the key Fe(−II) reactive intermediate species (**Int‐I**) is generated in situ from Fe_2_(CO)_9_ in the presence of CO, Cs_2_CO_3_, and the ligand. Although the precise formation pathway of the Fe(−II) species was not fully elucidated, it is proposed that the base and ligand jointly promote electronic reorganization and ligand exchange within the Fe_2_(CO)_9_ framework, leading to a low‐valent iron complex capable of undergoing a two‐electron transfer (TET) with the alkyl bromide to form **Int‐II**. Subsequent steps involve migratory CO insertion and alcohol nucleophilic attack, affording the desired ester product via acyl intermediates (**Int‐III** and **Int‐IV**). Reductive elimination from **Int‐IV** completes the catalytic cycle by regenerating an Fe(II) species.

**Scheme 12:**
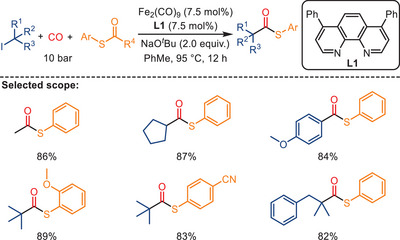
Iron‐catalyzed radical carbonylative thiolation of tertiary alkyl iodides with S‐aryl thioesters. 24 examples examined; yields: 77%–93%.

In 2024, Shao and co‐workers developed a related iron‐catalyzed radical carbonylation for the efficient synthesis of tertiary alkyl thioesters. These products are valuable intermediates in medicinal and biological chemistry (Scheme 12).^[^ ^78^ ^]^ The reaction employs Fe_2_(CO)_9_ as the catalyst precursor in combination with a phenanthroline ligand, under 10 bar of CO. Using S‐aryl thioesters as odorless sulfur sources, this method enables the coupling of unactivated tertiary alkyl iodides to afford thioesters in good‐to‐excellent yields. A wide range of functionalized alkyl iodides were tolerated, including those bearing esters, ethers, and even complex natural product fragments such as cholesterol, demonstrating the system's potential for late‐stage derivatization and sterically demanding substrate compatibility.

**Scheme 13:**
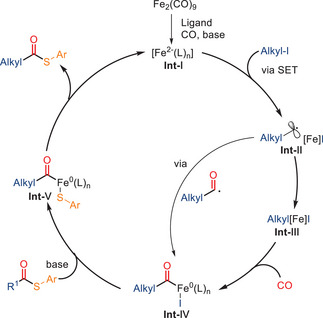
Proposed mechanism for the Fe‐catalyzed synthesis of tertiary alkyl thioesters from alkyl iodides.

In analogy to their previous work,^[^ ^77^ ^]^ the authors propose that the catalytically active Fe(−II) species are likely formed in situ from Fe_2_(CO)_9_ under the reaction conditions, with the phenanthroline ligand and the base promoting ligand exchange and electronic reorganization. Mechanistic studies indicate that the transformation proceeds via a single‐electron transfer (SET) pathway (Scheme 13). The reaction is initiated by the generation of tertiary alkyl radicals from alkyl iodides, which subsequently undergo CO insertion to yield acyl radicals. These acyl intermediates then couple with the S‐aryl thioesters, likely via an outer‐sphere radical substitution mechanism, leading to the formation of the corresponding tertiary alkyl thioesters. Radical trapping experiments with 2,2,6,6‐tetramethylpiperidin‐1‐oxyl (TEMPO) provided evidence for the radical nature of the process, and the use of structurally hindered substrates further supports a stepwise radical relay mechanism.

**Scheme 14:**
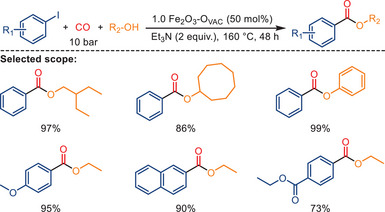
Fe‐catalyzed alkoxycarbonylation of aryl halides with alcohols. 27 examples examined; yields: 73%–99%.

**Figure 2:**
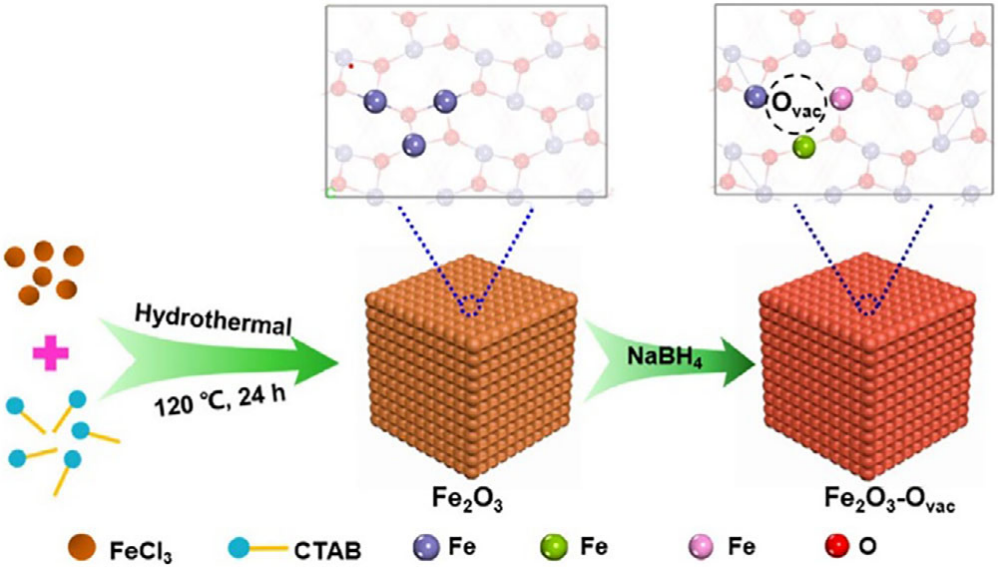
The procedure for the synthesis of the Fe_2_O_3_–O_vac_ catalysts. Reproduced with permission from Ref. [79]. Copyright 2023, Springer Nature.

Alkyl halides are typically more reactive toward nucleophiles than aryl halides. As a result, alkyl halides tend to undergo various substitution and elimination reactions more readily than aryl halides. Nevertheless, Fe‐catalyzed alkoxycarbonylations of aryl halides have been described recently. For example, in 2023, the Liu group reported a stimulating study about oxygen‐vacancy‐enriched Fe_2_O_3_ (Fe_2_O_3_‐Ovac) catalyzed carbonylation of various aryl halides and alcohols under CO atmosphere (Scheme 14).^[^ ^79^ ^]^ For this purpose, heterogeneous Fe_2_O_3_ materials with oxygen vacancies were synthesized by NaBH_4_ reduction of hydrothermally prepared Fe_2_O_3_. The content of oxygen vacancies increased gradually with the use of larger amounts of NaBH_4_. The increase in oxygen vacancies is believed to enhance the electronic density of surface Fe species in the catalyst, thereby improving catalytic activity (Figure 2). Reacting aryl halides (1.0 mmol) with alcohols (3.0 mL) and 10 bar of CO in the presence of Fe_2_O_3_–O_vac_ (80 mg, 50 mol%) furnished esters in good to excellent yields (73%–99% yield) under the optimal conditions (2 equiv. Et_3_N, 160 °C). The added triethylamine is necessary for the reaction to neutralize the hydrogen iodide formed during the reaction. Notably, the heterogeneous Fe_2_O_3_ catalyst with oxygen vacancies was reused five times without significant loss of activity, and the oxygen vacancy content remained nearly unchanged after recycling, indicating good structural stability of the material.^[^ ^79^ ^]^

## 3 Iron‐Catalyzed Carbonylation to Synthesize Amides

Amides are prevalent structural components in nature due to their fundamental role as the core linkage in natural proteins and peptides. This moiety is also present in numerous pharmaceuticals, polymers, agrochemicals, and fine chemicals. It is noteworthy that amide bonds exist in approximately 70% of the leading small‐molecule drugs among the top 50 best‐selling drugs in 2018.^[^ ^80^, ^81^ ^]^ Aminocarbonylation reactions, both intra‐ and intermolecular ones, represent effective methodologies for synthesizing amide derivatives. As substrates, halogenated hydrocarbons or alkynes easily react with carbon monoxide as a C_1_ feedstock, and amines as nucleophiles.^[^ ^82^, ^83^, ^84^, ^85^, ^86^ ^]^

**Scheme 15:**
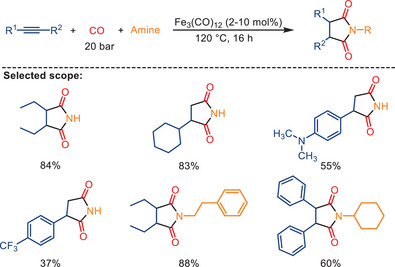
Reaction of various substituted alkynes with different amines. 15 examples examined; yields: 37%–88%.

In 2009, our group published the first report on the iron‐catalyzed synthesis of succinimides through double carbonylation of various terminal and internal alkynes in the presence of ammonia or amines, achieving high selectivity and activity (Scheme 15).^[^ ^87^ ^]^ The reaction was carried out under 20 bar of CO, with catalytic amounts of either [Fe(CO)_5_] or [Fe_3_(CO)_12_] serving as the catalyst and THF as the solvent. Symmetrical and nonsymmetrical aliphatic‐substituted, cyclic aliphatic‐substituted, and aromatic‐substituted succinimides were successfully obtained in the absence of any ligand. Electron‐donating substituents exhibited no effect on product yield, while electron‐withdrawing substituents resulted in reduced yields.

**Scheme 16:**
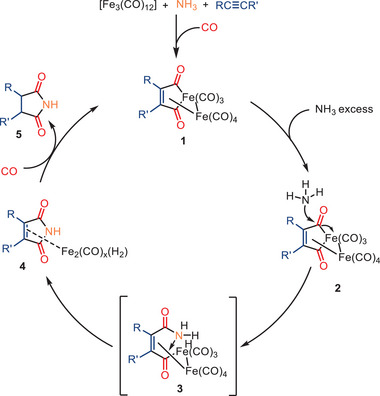
Proposed mechanism for the iron‐catalyzed double carbonylation of alkynes with amines.

The mechanism proposed for the iron‐catalyzed dicarbonylation of alkynes is shown in Scheme 16. As a first step, the amine reacts with [Fe_3_(CO)_12_] to give an amine‐coordinated iron carbonyl species, which then reacts with the alkyne to provide a metallacyclic intermediate, e.g., **1**. Although another hydrogenated complex was mentioned in the original proposal, it is unclear how this species reacts further on. Complex **1** is expected to react with NH_3_ to form intermediate **2**, which undergoes CO insertion to form the acyl species **3**. Subsequent intramolecular cyclization and reductive elimination afford intermediate **4**, which features an iron‐coordinated succinimide framework and an iron hydride species formed via proton or hydride transfer. The final conversion to succinimide **5** likely proceeds via elimination of a transient maleimide intermediate from **4**, followed by rapid hydrogenation in the presence of iron hydride species. Notably, the sequence in which reagents are introduced during the process has been demonstrated to exert a significant influence on the resulting product yield. The addition of amines to the catalyst solution prior to the introduction of substituted alkynes has been shown to result in an approximate 10% increase in yield. Furthermore, regeneration of intermediate **1** from **4** requires re‐entry of both CO and the alkyne, which were not explicitly depicted in the original mechanism.

**Scheme 17:**
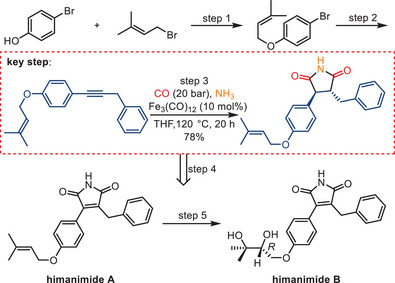
Iron‐catalyzed aminocarbonylation of alkynes as a key step in the short and efficient syntheses of himanimide A and B.

One year later, this methodology was applied as a key step in the total synthesis of himanimide A **1** and B **6**, which were originally isolated through fermentation from a *Serpula himantoides* strain collected in Chile by Prateeptongkum and colleagues (Scheme 17).^[^ ^88^ ^]^ This iron‐catalyzed carbonylation synthesis strategy can be expanded toward the total synthesis of related bioactive compounds, including camphorataimides, antrocinnamomins, polycitrins, and arcyriarubins.

**Scheme 18:**
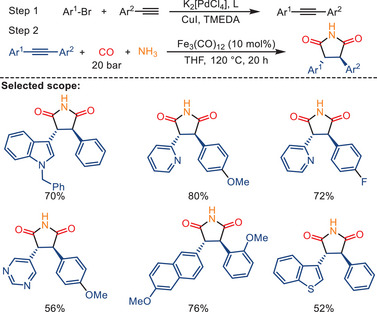
Sequential synthesis of 1,2‐diarylalkynes and trans‐3,4‐diarylsuccinimides. 20 examples examined; yields: 33%–85%.

In 2010, our group introduced minor modifications of the reaction conditions, allowing the use of more challenging unsymmetrical 1,2‐diarylalkynes or heteroaryl‐substituted alkynes in this double carbonylation process.^[^ ^89^ ^]^ As shown in Scheme 18, specifically the combination of palladium‐catalyzed Sonogashira reactions (step 1) with iron‐catalyzed double carbonylation (step 2) offers an efficient pathway to access various bioactive 3,4‐diaryl‐substituted succinimides and maleimides. In this work, the double aminocarbonylations of heterocyclic substrates using iron catalysts have been achieved for the first time.

**Scheme 19:**
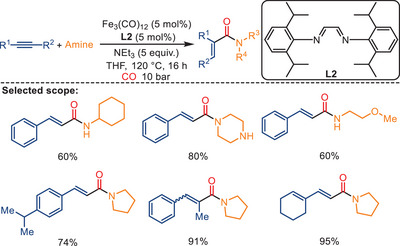
Iron‐catalyzed mono‐aminocarbonylation of alkynes. 19 examples examined; yields: 47%–96%.

Interestingly, using lower CO pressure (10 bar) in the presence of NEt_3_ (TEA), catalytic amounts of Fe_3_(CO)_12_, and **L3**, as opposed to the conventional double carbonylation, results in a mono‐carbonylation reaction of alkynes and amines leading to *α*,*β*‐unsaturated amides (Scheme 19).^[^ ^90^ ^]^ This general and selective iron‐catalyzed mono‐carbonylation provides a variety of cinnamides and acrylamides smoothly in good to very good yields. Moreover, the observed anti‐Markovnikov regioselectivity catalyzed by iron differs from the most known palladium‐catalyzed aminocarbonylation of alkynes.

**Scheme 20:**
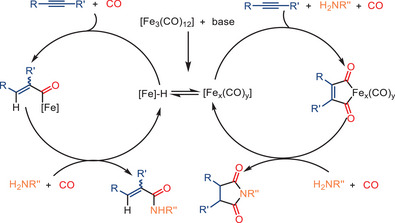
Proposed mechanism for the iron‐catalyzed mono‐ and double carbonylation of alkynes.

The plausible mechanism for mono‐ and double carbonylation reaction pathways is shown in Scheme 20. The formation of *α*,*β*‐unsaturated amides is proposed to proceed via a stepwise mechanism, while the double aminocarbonylation occurs through a concerted double insertion of CO. It is speculated that in the mono‐carbonylation process, the addition of the NEt_3_ to Fe_3_(CO)_12_ assists in forming [HNEt_3_][HFe_3_(CO)_11_]. This iron hydride carbonyl cluster then reacts with the alkyne. After formation of an acylcarbonyliron complex, the nucleophilic attack by the amine is expected to yield the corresponding acrylamide, while simultaneously regenerating the active iron species.

**Scheme 21:**
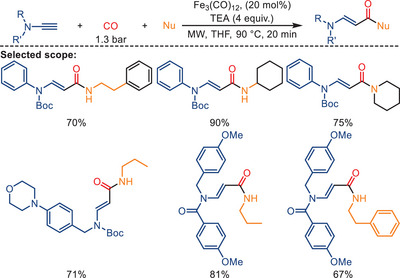
Iron‐catalyzed aminocarbonylation of ynamides by Petricci and co‐workers. 14 examples examined; yields: 44%–90%.

In the same year, Pizzetti's group reported the carbonylation of ynamides using microwave irradiation under low CO pressure (1.3 bar) (Scheme 21).^[^ ^66^ ^]^ The reactions were carried out using Fe_3_(CO)_12_ as both the catalyst and the source of carbon monoxide, with TEA serving as base or ligand. As expected, using microwave irradiation significantly reduces reaction times in this protocol. At higher pressure of CO the catalyst activity is reduced, resulting in lower product yields.

**Scheme 22:**
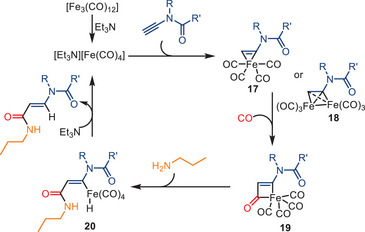
Proposed mechanism for the iron‐catalyzed monocarbonylation of ynamides.

A plausible mechanism of this carbonylation of ynamides reaction is presented in Scheme 22. The process is initiated by forming the active “amine‐[Fe(CO)_4_]” complex. After coordination with the ynamide, the intermediate **17** (or more plausibly, the dimer iron complex **18**) is formed. Following the insertion of CO into the Fe─C bond, the resulting complex undergoes nucleophilic attack by *n*PrNH_2_ with concurrent transfer of a hydride to the Fe center. The addition of TEA recreates the active [Et_3_N][Fe(CO)_4_] complex, releasing the product (*E*)‐acrylamide **21**. The carbamate group's potential assisting effect might prevent the formation of the succinimide derivative in this process.

**Scheme 23:**
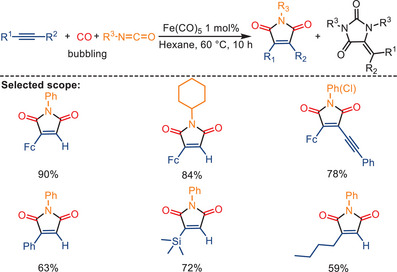
Fe(CO)_5_‐catalyzed formation of maleimides from alkynes, CO, and isocyanates by Mathur and co‐workers. 11 examples examined; yields: 25%–90%.

**Scheme 24:**
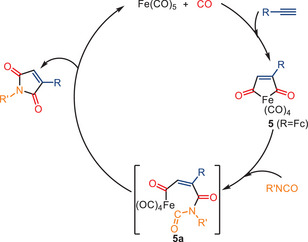
Proposed mechanism for the formation of maleimide according to Mathur and co‐workers.

In 2012, the Mathur group developed an interesting Fe(CO)_5_‐catalyzed [2 + 2 + 1] co‐cyclization, leading to pharmacologically important aryl/alkyl‐substituted maleimides and hydantoins from acetylene, isocyanate, and CO (Scheme 23).^[^ ^91^ ^]^ Under a CO atmosphere, the reaction yields maleimides as the primary products, achieving up to a 90% yield. Conversely, in the absence of CO, the reaction favors the formation of hydantoins, yielding the latter products in up to 87% yield. A plausible mechanism, supported by mechanistic experiments and existing literature data, is shown in Scheme 24.^[^ ^92^, ^93^ ^]^ The intermediate ferrole [{Fe(CO)_4_}(CO)_2_(FcC_2_H)] complex **5** is initially formed and has been successfully isolated. Subsequent insertion of an isocyanate into the Fe‐C bond of **5** led to the formation of a seven‐membered cyclic intermediate **5a**. This intermediate **5a** underwent reductive elimination to yield the corresponding maleimide, thereby regenerating the Fe(CO)_5_ catalyst.

In 2017, the same research group published an extension of their original work describing an Fe(CO)_5_‐catalyzed cycloaddition of alkynes with carbodiimide and CO under photolytic conditions to synthesize 5‐iminopyrrolones in moderate to good yields.^[^ ^94^ ^]^

**Scheme 25:**
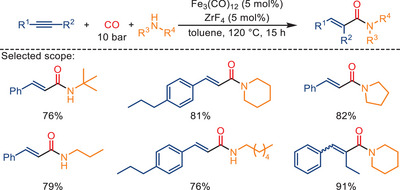
Fe‐catalyzed aminocarbonylation of different alkynes with different amines in the presence of ZrF_4_ as promotor. 25 examples examined; yields: 65%–93%.

**Scheme 26:**
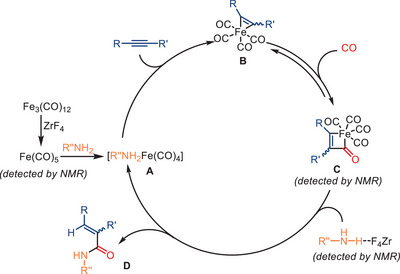
Proposed mechanism for the ZrF_4_‐promoted Fe‐catalyzed aminocarbonylation of alkynes.

Two years later, Huang and colleagues reported an unusual effect of zirconium fluoride in iron‐catalyzed aminocarbonylation reactions (Scheme 25).^[^ ^95^ ^]^ In this study, a catalytic amount of ZrF_4_ was effectively employed to promote the aminocarbonylation between alkynes and amines without the need for additional ligands. This operationally simple protocol tolerates a wide range of aromatic and aliphatic alkynes, including internal alkynes substrates, and allows facile access to various *α,β*‐unsaturated amides in good‐to‐excellent yields. Mechanistic studies revealed that the initial formation of active iron pentacarbonyl Fe(CO)_5_ occurs in situ through the interaction between ZrF_4_ and Fe_3_(CO)_12_ (Scheme 26). Following this step, the amine interacts with Fe(CO)_5_, leading to the generation of the [R″NH_2_Fe(CO)_4_] complex. This latter iron complex reacts with the alkyne, forming an acylcarbonyl iron species. Further steps involve CO insertion and the reaction of ZrF_4_‐activated amine, giving the final carbonylation product. In general, both the ZrF_4_ and the iron carbonyl species are believed to cooperate in facilitating the hydride transfer process.

**Scheme 27:**
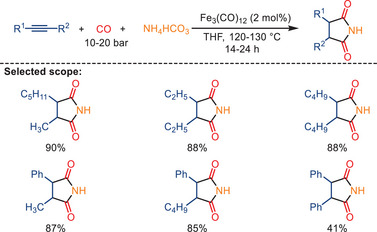
Fe_3_(CO)_12_‐catalyzed hydroaminocarbonylation of alkynes with NH_4_HCO_3_. 18 examples examined; yields: 21%–92%.

In 2021, Huang and colleagues developed another ligand‐free triiron dodecarbonyl‐catalyzed hydroaminocarbonylation of alkynes (Scheme 27).^[^ ^96^ ^]^ They utilized cheap and easy‐to‐handle NH_4_HCO_3_ as a solid ammonia source, avoiding the use of gaseous ammonia or liquid amines, for the preparation of succinimides. Under the optimal reaction conditions (alkyne: 0.5 mmol, CO: 10 bar, NH_4_HCO_3_: 5.0 mmol, Fe_3_(CO)_12_: 0.01 mmol, THF: 2.0 mL, 120 °C, 14 h), a wide range of aliphatic/aromatic internal and terminal alkynes reacted in an excellent manner with this catalytic system. Furthermore, the methodology was shown to be applicable for the gram‐scale (20 mmol) synthesis of succinimides using NH_4_HCO_3_.

**Scheme 28:**
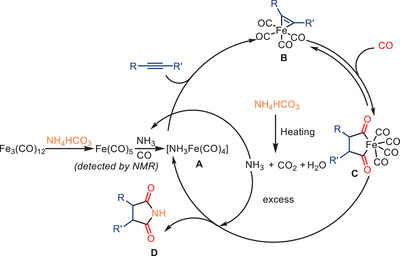
Proposed mechanism for the Fe‐catalyzed hydroaminocarbonylation of alkynes in the presence of NH_4_HCO_3_ to produce succinimides.

The proposed mechanism involves the conversion of the pre‐catalyst to active iron carbonyl Fe(CO)_5_, followed by the in situ generation of the iron carbonyl complex [NH_3_Fe(CO)_4_] **A** upon coordination with ammonia released from NH_4_HCO_3_ (Scheme 28). This coordination is believed to stabilize the active iron species by suppressing decomposition pathways. The alkyne subsequently reacts with the iron carbonyl intermediate **A** to form an alkyne‐iron carbonyl complex **B**, followed by CO coordination and insertion to generate the acyl‐Fe intermediate **C**. Nucleophilic attack by NH_3_ affords the final product **D**, regenerating the active iron species. According to the authors, NH_4_HCO_3_ not only provides a safe and convenient ammonia source but also contributes to the formation and stabilization of the catalytically active iron complex.

**Scheme 29:**
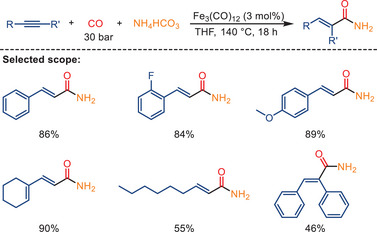
Fe‐catalyzed hydroaminocarbonylation of alkynes with NH_4_HCO_3_. 17 examples examined; yields: 46%–91%.

In 2022, Huang and co‐workers reported a related ligand‐free hydroaminocarbonylation of alkynes using NH_4_HCO_3_ (Scheme 29).^[^ ^97^ ^]^ In contrast to the work of the group of Deng, they used higher CO pressure (30 bar) and slightly higher temperatures. This combination resulted in a shift in the chemoselectivity of the carbonylation reactions. In lieu of double carbonylations, mono‐aminocarbonylation reactions occurred with a wide array of alkynes, encompassing aromatic alkynes, aliphatic alkynes, terminal alkynes, and internal alkynes. This process yielded linear *α,β*‐unsaturated primary amides in the presence of the same catalyst precursor (Fe_3_(CO)_12_). The electronic properties of substituents on the aromatic ring demonstrate a weaker influence on both reactivity and selectivity.

**Scheme 30:**
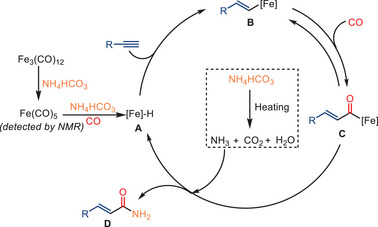
Proposed mechanism for the hydroaminocarbonylation of alkynes.

In agreement with the previous works, the formation of the active iron carbonyl Fe(CO)_5_ and iron hydride intermediate **A** are considered to be the key steps in the reaction mechanism. The steric hindrance of the terminal alkyne significantly influences the formation of the kinetically favored linear α,β‐unsaturated primary amides (Scheme 30).

**Scheme 31:**
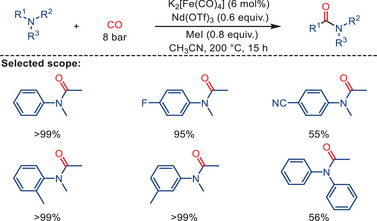
Direct Fe‐catalyzed carbonylation of C─N bonds in tertiary amines by Nasr Allah and co‐workers. 10 examples examined; yields: 28%–99%.

In general, the direct insertion of CO into existing C─N bonds can be regarded as an “ideal” method for synthesizing amides. Such protocols can streamline amide syntheses and save additional reaction steps. As a proof of concept, the Nasr Allah group developed the first iron catalysts for facilitating the insertion of CO into the C─N bonds of amines in 2019 (Scheme 31).^[^ ^98^ ^]^ More specifically, they used low‐valent iron complexes, such as Collman's reagent K_2_[Fe(CO)_4_], to facilitate the formation of amides from both aromatic and aliphatic amines in the presence of an iodoalkane promoter. The catalytic activity of the iron catalyst is significantly enhanced by inorganic Lewis acids like AlCl_3_ and Nd(OTf)_3_, enabling carbonylation to occur even at relatively low CO pressures (6–8 bars).

**Scheme 32:**
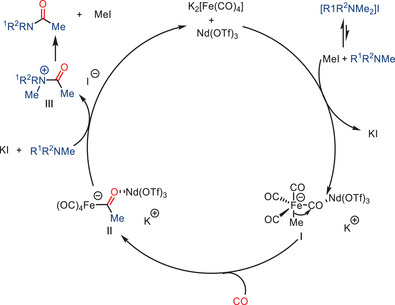
Proposed mechanism for the carbonylation of C─N bonds in tertiary amines.

According to the authors, in the proposed mechanism, alkylation of Collman's reagent K_2_[Fe(CO)_4_] with CH_3_I, which exists in an equilibrium with the amine reagent, produces the anionic alkyl complex [Fe(CH_3_)(CO)_4_]^−^ **I** (Scheme 32). Subsequent CO migratory insertion is promoted by the coordination of a Lewis acid co‐catalyst, leading to the formation of the acylferrate intermediate **II**. The free amine undergoes nucleophilic addition to the acyl ligand, resulting in the formation of the quaternary amide product **III**, followed by elimination. This process eventually yields the amide product while regenerating iodomethane. Notably, this mechanism avoids the activation of the strong C─N bond by initially generating an electrophilic acyl moiety. This moiety can then react with the amine, allowing the formation of the C─C bond between the alkyl residue and CO before cleaving the C─N bond.

**Scheme 33:**
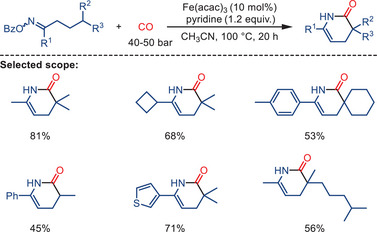
Iron‐catalyzed carbonylative cyclization of O‐benzoyl oxime esters to synthesize six‐membered lactams by Yin and co‐workers. 22 examples examined; yields: 40%–90%.

Several other novel approaches to the construction of amide derivatives through iron‐catalyzed processes were developed by Yin and co‐workers in recent years. In their first protocol,^[^ ^99^ ^]^ the key reaction step is a carbonylative cyclization of tertiary carbon radicals, which are generated through 1,5‐hydrogen atom transfer (HAT) from O‐benzoyl oxime esters (Scheme 33).

**Scheme 34:**
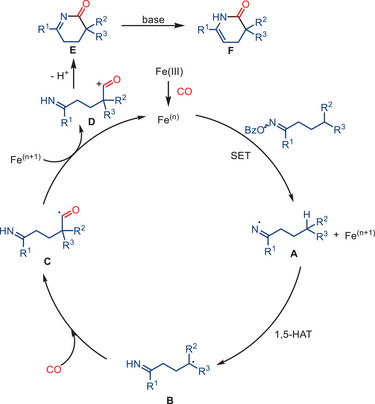
Mechanistic proposal for lactam formation via Fe‐catalyzed radical carbonylation.

A notable distinction emerges in this transformation, as Fe(acac)_3_ is utilized as the pre‐catalyst, a departure from the prevailing practices depicted in the preceding works using iron carbonyl complexes. Synthesis of structurally diverse six‐membered lactams is enabled under 40 bar CO pressure, yielding the corresponding products in moderate‐to‐excellent results. The method exhibits a broad substrate scope, including the possibility to use both cyclic and acyclic ketone‐derived oxime esters. It is compatible with various substituents or functional groups, such as halides, esters, and heterocycles. Mechanistic investigations suggest that the reaction proceeds via single‐electron transfer (SET) reduction of the oxime ester to form an iminyl radical, which then undergoes a 1,5‐HAT process to generate a more stable, sterically hindered tertiary carbon radical. This intermediate captures CO and undergoes intramolecular radical cyclization, yielding the corresponding lactam product. The proposed radical relay mechanism was supported by control experiments, radical trapping experiments, and structural analysis of key products (Scheme 34).

**Scheme 35:**
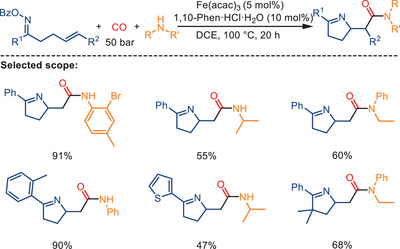
Fe‐catalyzed carbonylative cyclization of oxime esters with amines. 45 examples examined; yields: 39%–95%.

In 2020, another example employing a radical‐mediated carbonylative cyclization for amide synthesis has been reported by Wu and Zhang. Reacting *γ,δ*‐unsaturated aromatic oxime esters with amines under CO pressure, the authors prepared diverse *β*‐homoproline amide derivatives in the presence of Fe(acac)_3_ and 1,10‐phenanthroline (1,10‐Phen) hydrochloride as pre‐catalyst in dichloroethane as solvent (Scheme 35).^[^ ^100^ ^]^ It is suggested that the transformation proceeds via an iminyl radical‐triggered 1,5‐cyclization process, followed by intermolecular carbonylation enabled by the active iron species. This method tolerates a wide range of functional groups and enables access to structurally diverse nitrogen‐containing heterocycles in excellent yields. Moreover, the synthetic utility of the resulting carbonylated products is highlighted by their downstream conversion into hydrogenated or cyclized derivatives.

**Scheme 36:**
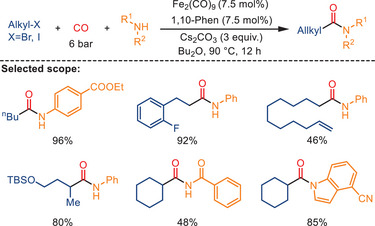
Iron‐catalyzed aminocarbonylation of unactivated alkyl halides. Over 100 examples examined; yields: 34%–100%.

In 2023, Ai and coworkers disclosed an iron‐catalyzed carbonylative reaction of alkyl bromides or alkyl iodides with amines, amides, and indoles, facilitating the synthesis of diverse carboxamides (Scheme 36).^[^ ^101^ ^]^ Employing Fe_2_(CO)_9_ as the pre‐catalyst, 1,10‐Phen as a ligand, and Cs_2_CO_3_ in Bu_2_O under 6 bar CO, unactivated alkyl halides underwent reaction with amines, amides, and indoles, providing the corresponding amides, imides, and N‐acyl indoles in good‐to‐excellent yields. The nature of the iron pre‐catalyst had a substantial impact on the carbonylative coupling reaction; only Fe° catalysts initiated the reaction successfully. No amide products were detected when Fe^2+^ or Fe^3+^ pre‐catalysts were employed. This method complements nicely well‐known noble metal‐catalyzed carbonylation reactions of alkyl halides.

**Scheme 37:**
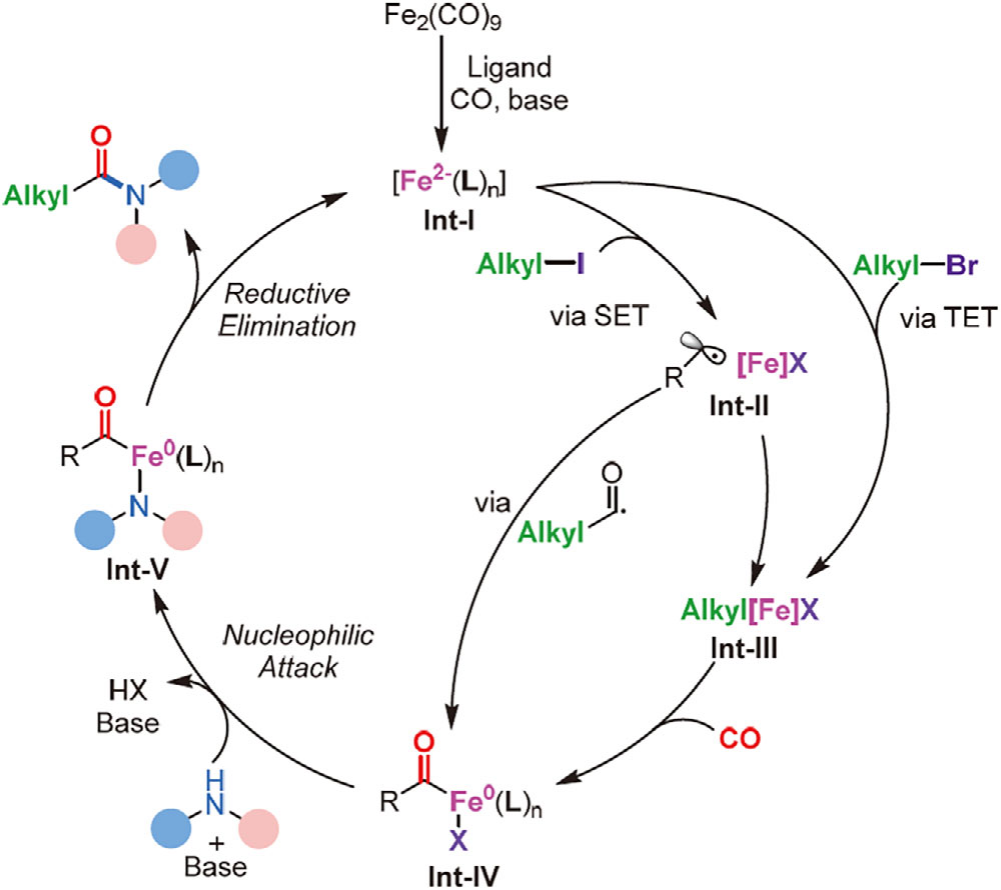
Proposed mechanism for the Fe‐catalyzed aminocarbonylation of unactivated alkyl halides. Reproduced with permission from Ref. [101]. Copyright 2023, Elsevier.

The authors proposed two different reaction pathways for alkyl iodides and alkyl bromides as substrates according to control experiments and related literature (Scheme 37). Firstly, the key species **Int‐I** is generated in the presence of a base under a CO atmosphere. Subsequently, it reacts with alkyl iodide via a single electron transfer (SET) process, resulting in the formation of the alkyl radical, which promptly interacts with the iron complex, yielding the iron alkyl species **Int‐III**. When alkyl bromide is used as the substrate, **Int‐III** is directly generated through a two‐electron transfer (TET) process, followed by CO migratory insertion. The resulting iron acyl complex **Int‐IV** may also originate from the in situ formed acyl radical. In the presence of the base, **Int‐IV** undergoes nucleophilic attack, yielding **Int‐V**. The overall catalytic conversion is then completed by reductive elimination, yielding the final product. Concurrently, the Fe^2−^ complex is regenerated for the subsequent catalytic cycle.

**Scheme 38:**
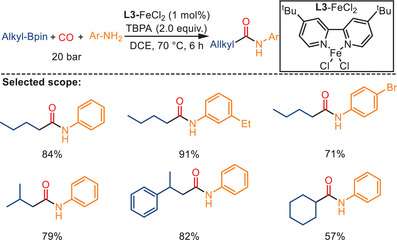
Iron‐catalyzed aminocarbonylation of alkylboronic esters with arylamines by Gu and co‐workers. 27 examples examined; yields: 47%–95%.

A complementary radical‐based aminocarbonylation strategy was further developed by Wu and co‐workers. More specifically, they employed alkylboronic pinacol esters (alkyl‐Bpin) as alkyl radical precursors in place of traditional alkyl halides (Scheme 38).^[^ ^102^ ^]^ In the presence of FeCl_2_ and 4,4′‐di‐tert‐butyl‐2,2′‐bipyridine (dtbbpy) as the catalyst and tert‐butyl peracetate (TBPA) as an oxidant, a broad range of alkyl‐Bpin derivatives reacted smoothly with arylamines under 20 bar CO, affording the corresponding aliphatic amides in moderate to excellent yields. The investigation by Gu and coworkers revealed that both primary and secondary alkyl‐Bpin substrates were well tolerated. Furthermore, the protocol was successfully scaled up to gram quantities, thereby demonstrating its practical utility.

**Scheme 39:**
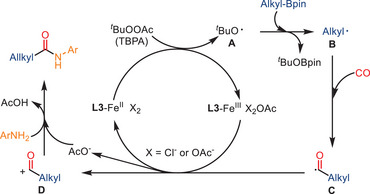
Proposed radical mechanism for the aminocarbonylation of alkylboronic esters.

The proposed mechanism involves an initial Fe(II)‐mediated homolysis of TBPA, generating tert‐butoxy radicals, which in turn form alkyl radicals. These latter radicals are subsequently captured by CO to generate the corresponding acyl radicals, which react with amines to form the final amide products (Scheme 39). Radical trapping experiments with TEMPO and 1,1‐diphenylethylene provided strong evidence for the involvement of alkyl and acyl radical intermediates. This method also highlights the potential of organoboron reagents as radical precursors in iron‐catalyzed carbonylation chemistry.

**Scheme 40:**
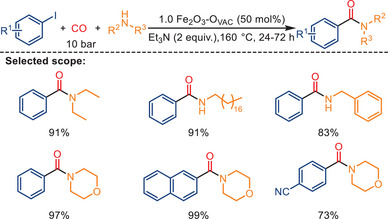
Heterogeneous Fe‐catalyzed carbonylation of aryl halides with amines by Liu and co‐workers. 33 examples examined; yields: 71%–99%.

**Scheme 41:**
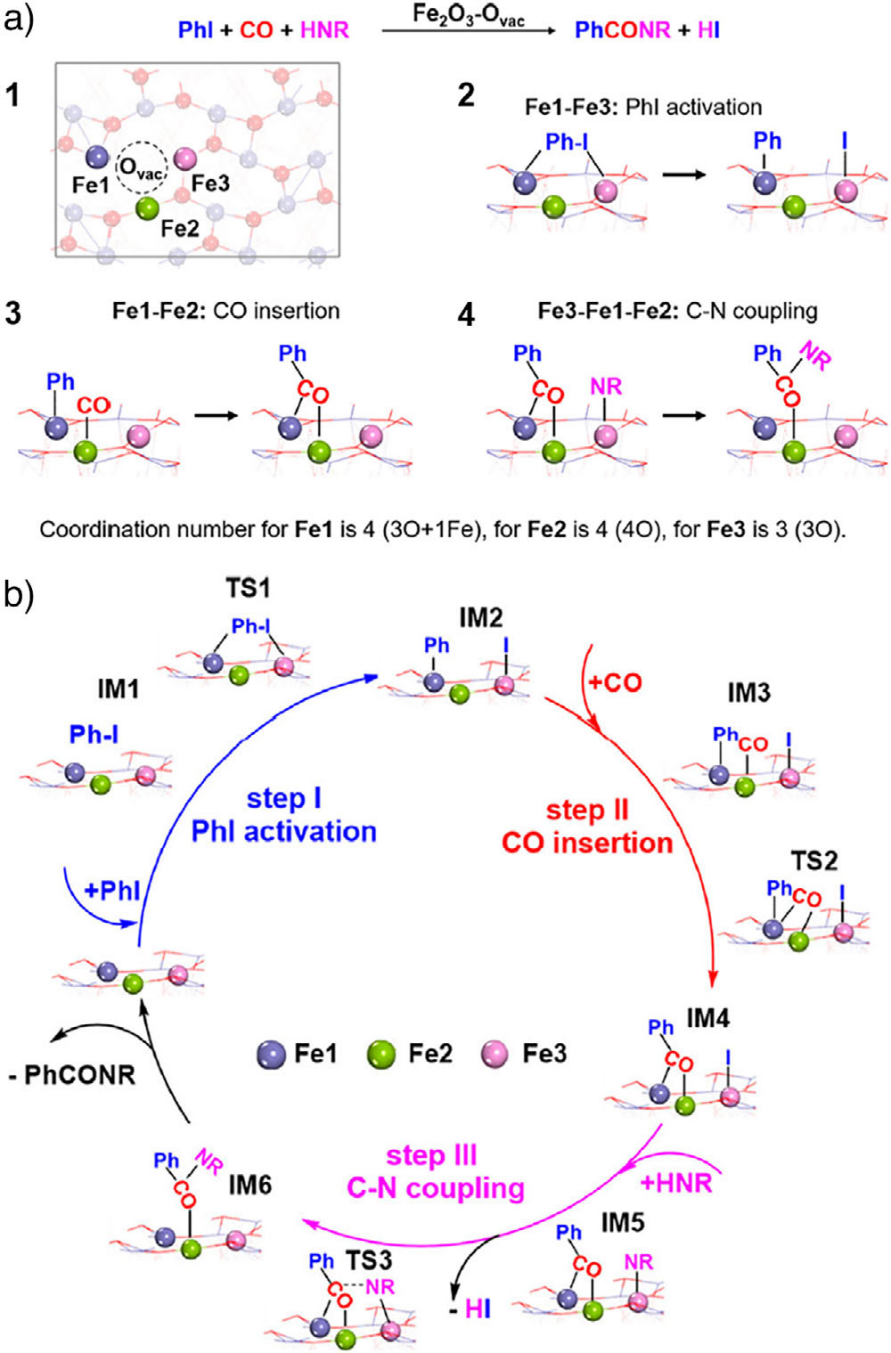
Proposed reaction pathway for the aminocarbonylation of iodobenzene in the presence of 1.0 Fe_2_O_3_–O_vac_. HNR denotes a secondary amine (RR′NH), as originally represented in the source. Reproduced with permission from Ref. [79]. Copyright 2023, Nature Publishing Group.

While the vast majority of carbonylation reactions in organic synthesis are performed in the presence of homogeneous catalysts, the application of heterogeneous catalysts in such reactions is scarce.^[^ ^103^, ^104^ ^]^ In this respect, the work of Liu and co‐workers is of considerable interest. The scope of their study expanded the conventional limits of aminocarbonylation of alkyl halides with CO beyond homogeneous Fe catalysis. For this purpose, they utilized a reusable Fe_2_O_3_–O_vac_ catalyst system (Scheme 40).^[^ ^79^ ^]^ This system effectively tolerated cyclic and aliphatic acyclic secondary amines with steric hindrance, as well as both aromatic amines and aliphatic, and cyclic primary amines, successfully converting them into the corresponding amides. The experimental studies combined with DFT calculations revealed the formation of **Fe1**, **Fe2**, and **Fe3** sites on Fe_2_O_3_–O_vac_. These sites have been shown to catalyze the elementary steps involved in iodobenzene activation, CO insertion, and C─N/C─O coupling, thereby significantly enhancing the carbonylation process (Scheme 41). In the proposed mechanism, iodobenzene is expected to adsorb and then activated on the surface of Fe_2_O_3_–O_vac_ (step **I**).

Subsequently, the C─I bond cleavage results in the spontaneous movement of the phenyl group from iodobenzene to the **Fe1** site, leading to the formation of the **IM2** intermediate. Then, CO insertion is initiated by CO adsorption on the **Fe2** site, followed by its insertion into the **Fe1**‐C bond, generating the acyl intermediate PhCO* (**IM4**) (step **II**). This **IM4** intermediate facilitates amide formation by starting with the adsorption of [HNR] on the **Fe3** site (**IM5**) (step **III**). Finally, iodine is displaced by NR*, leading to the formation of HI. NR* then attacks the C site of PhCO*, resulting in the corresponding amide product formation (**IM6**).

## 4 Iron‐Catalyzed Carbonylation to Synthesize Ketones, Aldehydes, and Carboxylic Acids

It is interesting to note that the majority of iron‐catalyzed carbonylation processes have been performed in the presence of amines. Aminocarbonylations are typically more straightforward to execute in comparison to related alkoxy‐ or hydroxycarbonylations. The impact of amines on the stability of active iron catalyst species and the facilitation of carbonylations under milder conditions is not yet fully elucidated. However, preliminary findings suggest a potential stabilization effect of amines, thereby enabling the occurrence of carbonylations. Nevertheless, alternative nucleophiles have been utilized, and such transformations offer potential for more general methodology developments. To initiate the investigation, the synthesis of ketones will be examined. Ketones and their derivatives play a crucial role as structural motifs in a variety of biologically active molecules and pharmaceuticals. They function as significant synthons in the synthesis of complex molecules.^[^ ^105^, ^106^, ^107^, ^108^ ^]^ A particularly appealing and effective method for synthesizing these molecules involves transition metal‐catalyzed carbonylation reactions, with CO as a C_1_ feedstock.

**Scheme 42:**
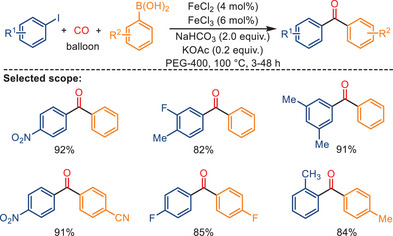
Iron‐catalyzed carbonylative Suzuki reactions by Zhong and Han. 38 examples examined; yields: 71%–97%.

In 2014, Zhong and Han reported an iron‐catalyzed carbonylative Suzuki reaction employing atmospheric pressure CO (Scheme 42).^[^ ^109^ ^]^ Surprisingly, the best results were obtained utilizing both FeCl_2_ and FeCl_3_ as the pre‐catalysts. Under optimal reaction conditions (PEG‐400 as solvent, NaHCO_3_ as the base, atmospheric pressure CO, 100 °C) aryl iodides reacted with arylboronic acids, giving the corresponding ketone products in 71%–97% yield. In addition, KOAc was employed as an external promoter to increase the product yield in this reaction. Various substituted diaryl ketones were successfully obtained in the absence of any ligand.

**Scheme 43:**
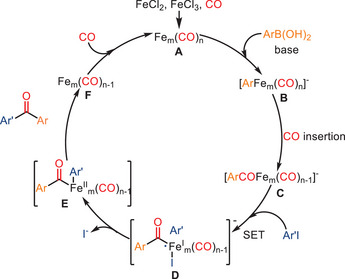
Proposed reaction pathway for iron‐catalyzed carbonylative Suzuki reactions.

In their mechanistic proposal (see Scheme 43), the authors suggest that the catalytic cycle begins with the interaction between FeCl_3_ and FeCl_2_ with CO, generating Fe*_m_* (CO)*_n_* (**A**) complexes.^[^ ^110^ ^]^ The arylboronic acid subsequently undergoes an addition reaction with **A**, forming a highly nucleophilic organoiron complex **B** with the assistance of base. This complex then undergoes intramolecular carbon monoxide migratory insertion, leading to the generation of organoiron complex **C**. After the S_N_R1‐type nucleophilic oxidative addition between **C** and ArI, organoiron complex **D** is formed. Finally, the desired carbonylated product is released through reductive elimination.

**Scheme 44:**
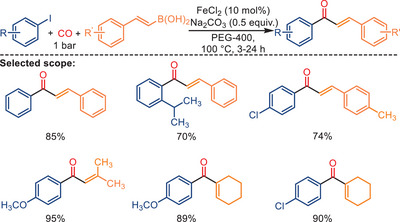
Ligand‐free Fe‐catalyzed carbonylative coupling of aryl iodides with alkenyl boronic acids. 37 examples examined; yields: 50%–95%.

Although the proposed mechanism involves the formation of Fe*_m_* (CO)*_n_* species from FeCl_2_ and FeCl_3_, it should be noted that no direct experimental evidence (e.g., spectroscopic characterization or isolation) was provided in the original study to confirm the generation of these iron carbonyl species. Instead, the authors conducted control experiments using Fe powder and Fe_3_(CO)_12_ as alternative catalysts, which resulted in lower product yields, suggesting that low‐valent iron species may be involved in the formation of the active catalyst. Nevertheless, the exact nature of the catalytically active species remains unclear and warrants further investigation. To expand the substrate scope beyond arylboronic acids, Xu and co‐workers reported a related ligand‐free iron‐catalyzed carbonylative coupling of aryl iodides with alkenyl boronic acids, enabling the synthesis of *α,β*‐unsaturated ketones under atmospheric CO pressure (Scheme 44).^[^ ^111^ ^]^ This method features operational simplicity, mild conditions, and the absence of ligands or precious metals, employing simply FeCl_2_ and Na_2_CO_3_ in PEG‐400 as the catalytic system. A wide range of aromatic and aliphatic alkenyl boronic acids were well tolerated in this process, affording the desired enones in good to excellent yields. The reaction also exhibited scalability, with gram‐scale transformations conducted successfully. Given the similarity in reaction design and conditions, the proposed mechanism is believed to follow a pathway analogous to that of the previously reported Suzuki‐type carbonylation, involving formation of a carbonyl iron species, subsequent migratory CO insertion, and reductive elimination after coupling with aryl iodides.

**Scheme 45:**
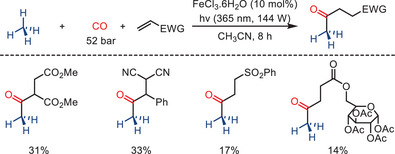
Fe‐photocatalyzed carbonylation of methane with carbon monoxide and alkenes yielding unsymmetrical ketones by Raymenants and co‐workers. 41 examples examined; yields: 14%–93%.

Due to the low reactivity of C─H bonds, direct C─H functionalization at sp^3^‐hybridized carbon atoms is challenging, especially for light alkanes.^[^ ^112^, ^113^, ^114^ ^]^ Thus, in the domain of carbon–hydrogen bond activation and functionalization (C─H activation/functionalization), numerous recent endeavors have been undertaken to catalyze the functionalization of C─H bonds in methane. The significant presence of methane in natural gas or shale gas deposits demonstrates its substantial potential for utilization as a feedstock in contemporary chemistry, especially for bulk‐scale applications. In this respect, the recent work of Raymenants and co‐workers is notable. In 2023, they described a continuous‐flow photocatalytic strategy that enables the direct iron‐catalyzed C(sp^3^)–H carbonylation of methane using a photocatalytic hydrogen atom transfer (HAT) in combination with CO (Scheme 45).^[^ ^103^ ^]^ The inexpensive and readily available FeCl_3_·6H_2_O was utilized as a HAT photocatalyst. Additionally, it acted as a source of Cl^•^, which serves as a powerful hydrogen abstractor. Using this protocol, dimethyl 2‐methylsuccinate, benzylidene malononitrile, benzyl vinyl sulfone, and the acrylate of an acetyl‐protected sugar were directly acylated with methane.

**Scheme 46:**
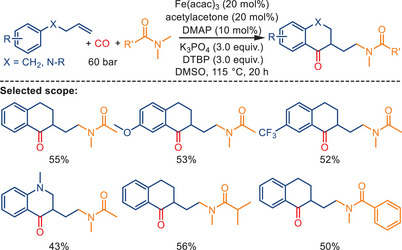
Iron‐catalyzed aminoalkylative carbonylative cyclization of alkenes with amides toward α‐tetralones by Sun and co‐workers. 18 examples examined; yields: 35%–64%.

Another strategy for ketone construction involves iron‐catalyzed carbonylative cyclization via radical relay. Sun and co‐workers reported an aminoalkylative carbonylative cyclization of 4‐aryl‐1‐butenes with amides under CO pressure. This process yields valuable ketone products, α‐tetralones, as shown in Scheme 46).^[^ ^115^ ^]^ The reaction employs Fe(acac)_3_ (20 mol%) as the catalyst and 4‐dimethylaminopyridine (DMAP) and acetylacetone as co‐ligands. It also uses di‐tert‐butyl peroxide (DTBP) and K_3_PO_4_ under 60 bar of CO. Applying this protocol, a broad substrate scope was demonstrated, with *ortho*‐, *meta*‐, and *para*‐substituted arylbutenes and diverse acetamides yielding the corresponding α‐tetralones in moderate yields. Functional groups and substituents such as methyl, methoxy, halogens, CF_3_, esters, and heteroatoms were well tolerated.

**Scheme 47:**
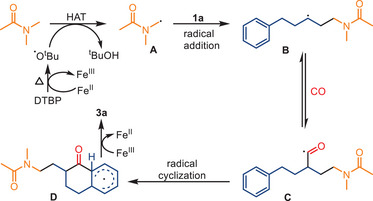
Proposed mechanism for the formation of α‐tetralones via aminoalkylative carbonylation.

In the proposed mechanism (Scheme 47), the reaction is initiated by the thermal decomposition of DTBP, which generates tert‐butoxy radicals. These radicals abstract a hydrogen atom from *N*,*N*‐dimethylacetamide, forming an *α*‐aminoalkyl radical **A**. Radical **A** subsequently adds to the alkene, resulting in the formation of alkyl radical **B**. This radical then captures CO, yielding an acyl radical **C**. The acyl radical undergoes intramolecular cyclization onto the aromatic ring, leading to the formation of the final product via single‐electron oxidation. Radical trapping experiments using TEMPO and 1,1‐diphenylethylene (DPE) support the involvement of radical intermediates in this transformation.

Aromatic aldehydes are valuable intermediates in organic synthesis, with a wide range of applications in the production of pharmaceuticals, agrochemicals, and functional materials. To access these compounds efficiently, iron‐catalyzed hydroformylation and reductive carbonylation reactions have been developed, offering interesting alternatives to traditional noble‐metal‐catalyzed processes.^[^ ^116^, ^117^ ^]^

**Scheme 48:**
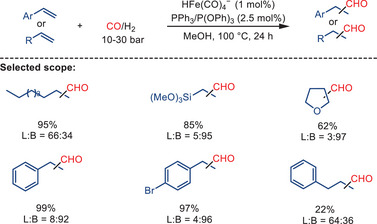
Iron‐catalyzed hydroformylation of alkenes by Pandey and co‐workers. L:B = linear‐to‐branched ratio. 21 examples examined; yields: 10%–99%.

**Scheme 49:**
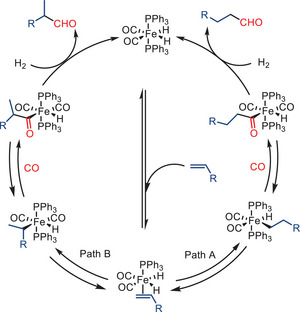
Proposed mechanism for iron‐catalyzed hydroformylation of alkenes.

As an example, Pandey and co‐workers reported an efficient iron‐catalyzed hydroformylation of alkenes under mild conditions (10–30 bar syngas pressure and temperatures below 100 °C), providing a variety of aldehydes with good to excellent yields (Scheme 48).^[^ ^44^ ^]^ According to the authors, the catalytic cycle is initiated by the in situ generation of iron dihydride complexes from [HFe(CO)_4_]^−^ in the presence of phosphine ligands and acetic acid (Scheme 49). The alkene coordinates with the iron center, followed by a migratory insertion into the Fe─H bond, generating an alkyl‐iron intermediate. Subsequently, CO insertion leads to the formation of an acyl–iron complex, which undergoes reductive elimination to release the desired aldehyde product. The authors also demonstrated that the addition of acetic acid has a significant impact on the rate of the reaction, which is attributed to the enhancement of the generation of Fe─H species. These Fe─H species have been identified as being crucial for the hydride transfer step.

**Scheme 50:**
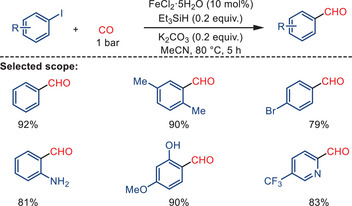
Iron‐catalyzed reductive carbonylation of aryl iodides to afford aromatic aldehydes by Akkineni and co‐workers. 18 examples examined; yields: 71%–92%.

Building upon ligand‐free systems, Akkineni and co‐workers further developed a simple and practical iron‐catalyzed reductive carbonylation of aryl iodides to afford aromatic aldehydes under ambient CO pressure (Scheme 50).^[^ ^118^ ^]^ Using FeCl_2_·5H_2_O as the pre‐catalyst, triethylsilane as the reducing agent, and K_2_CO_3_ as the base in acetonitrile at 80 °C, a wide variety of substituted aryl iodides were transformed into the corresponding aldehydes in moderate to excellent yields. The protocol operates without the need for additional ligands and tolerates electron‐donating and electron‐withdrawing substituents, providing a convenient and scalable approach to aromatic aldehydes.

In general, the carbonylative synthesis of carboxylic acids (hydroxycarbonylation) is closely related to the aforementioned alkoxy‐ and aminocarbonylation processes. The primary distinction between these methodologies lies in the utilization of water as a nucleophile, as opposed to amines or alcohols, which presents a series of challenges for the catalytic system. Carboxylic acids are of significant interest in the fields of (bio)chemistry and polymer chemistry. Consequently, researchers are continually developing direct and effective methods for carboxylation with carbon monoxide.^[^ ^119^, ^120^, ^121^ ^]^

**Scheme 51:**
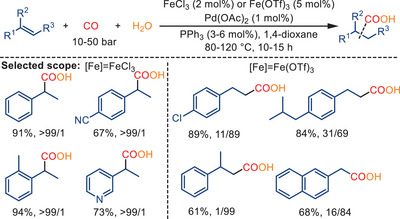
Palladium‐catalyzed hydroxycarbonylation of olefins in the presence of FeCl_3_ or Fe(OTf)_3_. 44 examples examined; yields: 45%–96%.

**Scheme 52:**
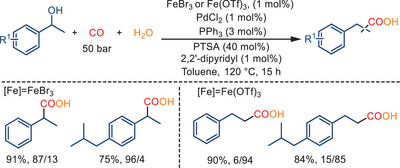
Palladium‐catalyzed hydroxycarbonylation of 1‐arylalcohols in the presence of FeBr_3_ or Fe(OTf)_3_. 4 examples examined; yields: 75%–91%.

Although palladium serves as the main catalyst species in the following cases, they are included here to underscore the important promoting role of Fe(III) salts in hydroxycarbonylation reactions. In 2018, Huang and colleagues reported the promoting effect of Fe(III) salts in Pd‐catalyzed hydroxycarbonylations of alkenes and alcohols, as depicted in Schemes 51 and 52.^[^ ^122^ ^]^ The hydroxycarbonylation of both aromatic and aliphatic alkenes is significantly enhanced in terms of reactivity and selectivity (*iso/n* or *n/iso* up to >99:1) by employing FeCl_3_ or Fe(OTf)_3_ (Scheme 51). The choice of the anion significantly influences the styrene insertion step, which in turn is crucial in determining the regioselectivity of the reaction.

When employing FeBr_3_ as the promoter for the carbonylation of 1‐arylethanols, branched carboxylic acids are mainly generated with 87%–96% regioselectivity. Conversely, the use of Fe(OTf)_3_ resulted in the formation of the corresponding linear products with up to 94% regioselectivity (Scheme 52). In both processes, Fe(III) salts serve as effective Lewis acids and co‐catalysts, significantly enhancing the hydroxycarbonylations reaction. It was proposed that the hydrolysis of Fe(III) salts in the presence of water creates an “acidic‐buffer system” that facilitates the formation of Pd─H. Importantly, the reaction failed to occur in the absence of Fe(III) salts.

**Scheme 53:**
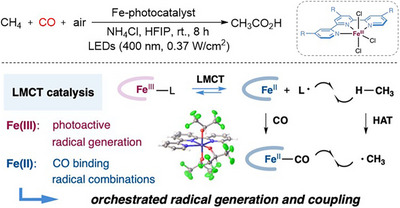
Photocatalytic aerobic oxidative carbonylation of methane by iron terpyridine catalysts, reported by Liu and Zuo and co‐workers. HFIP = hexafluoroisopropanol. Reproduced with permission from Ref. [127]. Copyright 2025, American Chemical Society.

Apart from the hydroxycarbonylation of olefins or activated C─X (X = halide, OH), the catalytic oxidative carbonylation of C─H bonds is a subject of considerable interest. The prime example of such a reaction is the direct carboxylation of methane, which is regarded as the optimal method for a sustainable acetic acid synthesis.^[^ ^123^, ^124^, ^125^ ^]^ Significant progress in this highly challenging area has recently been reported by Zuo and co‐workers, who developed a photocatalytic protocol employing Fe(III)‐terpyridine complexes for the aerobic carbonylation of methane into acetic acid under visible light irradiation (Scheme 53).^[^ ^126^, ^127^ ^]^ Notably, this transformation proceeds at room temperature under CO and air, thereby circumventing the safety problems of CO–O_2_ mixtures, delivering acetic acid with outstanding C_2_/C_1_ selectivity (up to 20:1) and an exceptional photocatalyst turnover number (TON) up to 10 680. Moreover, the protocol has been successfully extended to other light alkanes, including ethane, propane, butane, pentane, and norbornane. This development enables the direct conversion to their corresponding carboxylic acid homologs with high selectivity. Unfortunately, so far, this protocol has only been executed on an mg‐scale (1 µmol of Fe catalyst). However, if a scale‐up can be accomplished in the future, it will offer many intriguing practical applications. The authors propose that the reaction is initiated via ligand‐to‐metal charge transfer (LMCT) excitation, in which the Fe(III)–terpyridine complex generates an Fe(II) species and heteroatom‐centered radicals. These radicals abstract a hydrogen radical from methane, producing methyl radicals, which are subsequently trapped by Fe(II)–CO complexes to form the corresponding acyl radical intermediates. The latter are then oxidized to generate acetic acid. Mechanistic insights from radical inhibition experiments, isotope labeling, and DFT calculations support a radical relay pathway involving Fe‐centered activation and outer‐sphere CO insertion.

**Scheme 54:**
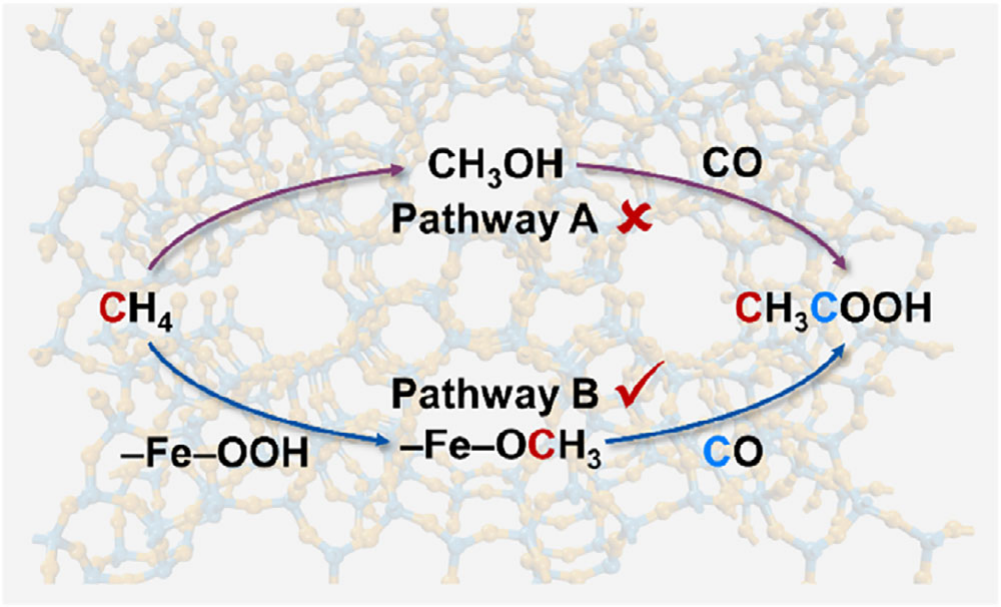
The possible reaction pathways for the formation of acetic acid from methane and carbon monoxide according to Wang and co‐workers. Reproduced with permission from Ref. [128]. Copyright 2023, Elsevier.

Another example of the synthesis of acetic acid from methane and carbon monoxide using iron catalysts was reported by the Wang group in 2023.^[^ ^128^ ^]^ In the presence of an Fe/ZSM‐5 (0.25) zeolite catalyst, prepared by impregnation method, methane reacted with carbon monoxide and hydrogen peroxide to give acetic acid at a temperature range of 25–75 °C. The mechanism suggests that Fe–OCH_3_ species serve as plausible intermediates, and mononuclear Fe^3+^ species play a crucial role in achieving the relatively high yield (Scheme 54).

## 5 Summary and Outlook

In the past 25 years, the use of iron‐based complexes and salts in organic synthesis and homogeneous catalysis has increased exponentially, becoming a subject of significant interest for researchers worldwide. In this context, iron‐catalyzed carbonylation methodologies have seen a renaissance, too. Even though the initial instances of such processes were documented over seven decades ago, it was not until the advent of the 21st century that a significant number of carbonylation reactions were developed. These reactions sought to supplant the use of rare, costly, and frequently toxic noble metal catalysts with the more abundant element iron. In fact, applying iron catalysts for valorization of readily available feedstocks with carbon monoxide (CO) gas facilitates the synthesis of a multitude of valuable carbonyl compounds, including ketones, carboxylic acids, esters, and amides. A prominent example is the widely investigated carbonylation of alkynes, which can be regarded as a standard methodology in the repertoire of synthetic organic chemists. These multicomponent carbonylative reactions have demonstrated enhanced flexibility and step economy when compared to alternative traditional synthetic methods. Furthermore, in recent years, significant advancements have been made in novel transformations, including radical carbonylations, the direct C(sp^3^)–H carbonylation of alkanes, and the oxidative carbonylation of methane.

Despite the numerous advantages and advancements in the field, as well as the continuous efforts made towards the development of more active and productive iron (pre‐)catalysts, the practicality of many reported methods for applications in the industrial fine and bulk chemical synthesis remains a challenge. In this regard, the following key challenges must be addressed in the future:

- Improving activity levels of the respective catalysts
- Tolerance of the developed methods against impurities and better robustness of the process conditions.
- It is imperative to ascertain the “authentic” catalytically active species, as such mechanistic discernment will not only facilitate the rational development of iron catalysts but also establish the foundation for unanticipated breakthroughs in this domain.
- From an academic perspective, the role of iron catalysis in carbonylation reactions necessitates rigorous substantiation, particularly in the context of excluding the potential impact of noble metal impurities present in the feedstocks or within the autoclave apparatus under carbon monoxide (CO) pressure.

We are confident that these goals can be achieved and that selected Fe‐catalyzed reactions will achieve comparable efficacy to those catalyzed by precious metals within the next decade. Finally, it is hoped that this review will draw more attention from the catalysis community to iron‐catalyzed carbonylation reactions and inspire synthetic organic chemists to incorporate these innovative methods as valuable tools in their synthetic chemistry toolbox.

## Conflict of Interests

The authors declare no conflict of interest.

## Data Availability

Data sharing is not applicable to this article as no new data were created or analyzed in this study.
